# Polyunsaturated fatty acids and calcaneal ultrasound parameters among Inuit women from Nuuk (Greenland): a longitudinal study

**DOI:** 10.3402/ijch.v72i0.20988

**Published:** 2013-06-05

**Authors:** Alexandra-Cristina Paunescu, Pierre Ayotte, Éric Dewailly, Sylvie Dodin, Henning S. Pedersen, Gert Mulvad, Suzanne Côté

**Affiliations:** 1 Axe santé publique et pratiques optimales en santé, Centre de Recherche du CHU de Québec, Québec, Canada; 2 Laboratoire de toxicologie, Institut national de santé publique du Québec, Québec, Canada; 3 Medical Centre, Dronning Ingrids Hospital, Nuuk, Greenland; 4 Center of Primary Health Care, Queen Ingrid's Hospital, Nuuk, Greenland; 5 Greenland Center for Health Research, University of Greenland, Nuuk, Greenland

**Keywords:** Inuit women, Greenland, calcaneal ultrasound parameters, polyunsaturated fatty acids

## Abstract

**Background:**

The traditional diet of Inuit people comprises large amounts of fish and marine mammals that are rich in omega-3 polyunsaturated fatty acids (PUFAs). Results from *in vitro* studies, laboratory animal experiments and population studies suggest that omega-3 PUFA intake and a high omega-3/omega-6 ratio exert a positive effect on bone health.

**Objective:**

This longitudinal study was conducted to examine the relationship between omega-3 and omega-6 PUFA status and quantitative ultrasound (QUS) parameters in Greenlandic Inuit women.

**Methods:**

The study included 118 Inuit women from Nuuk (Greenland), aged 49–64 years, whose QUS parameters measured at baseline (year 2000), along with PUFA status and covariates, and follow-up QUS measurements 2 years later (year 2002). QUS parameters [speed of sound (SOS); broadband ultrasound attenuation (BUA)] were measured at the right calcaneus with a water-bath Lunar Achilles instrument. Omega-3 and omega-6 PUFA contents of erythrocyte membrane phospholipids were measured after transmethylation by gas chromatography coupled with a flame ionization detector. Relationships between QUS parameters and different PUFAs were studied in multiple linear regression models.

**Results:**

Increasing values of EPA, DHA and the omega-3/omega-6 PUFA ratio were associated with increased BUA values measured at follow-up (year 2002). These associations were still present in models adjusted for several confounders and covariates. We found little evidence of associations between PUFAs and SOS values.

**Conclusion:**

The omega-3 PUFA intake from marine food consumption seems to have a positive effect on bone intrinsic quality and strength, as revealed by higher BUA values in this group of Greenlandic Inuit women.

Inuit people living in circumpolar regions are submitted to specific geographic and climatic conditions and display unique lifestyle habits, most notably their traditional diet based on local game and marine species harvesting. Inuit consume large amounts of fatty fish and marine mammal fat (1), which explain their higher omega-3 polyunsaturated fatty acid (PUFA) intake compared to that of non-Aboriginal populations (2).

Intakes of omega-3 and omega-6 PUFAs in balanced proportions seem essential for proper cellular function and the prevention of inflammatory, autoimmune and cardiovascular diseases as well as diabetes and obesity (3). Both PUFA families are also involved in bone metabolism, but their optimal proportion for bone health has yet to be identified (4).

In rats, omega-3 PUFAs induce positive effects on bone strength (5), bone mass and the microarchitecture of long bones (6), bone mineral content (BMC) and bone remodelling (7), whereas improved mineralization and bone mineral density (BMD) were noted in vertebrae and femur of transgenic mice fat-1 (8). Some studies in laboratory animals mainly attributed these effects to docosahexaenoic acid (DHA) (7–9), alpha linolenic acid (ALA) and eicosapentaenoic acid to (EPA) (9). *In vitro* and laboratory animal studies also showed that a diet with a low omega-6/omega-3 ratio may have a beneficial effect on BMC (10) or BMD (4).

In humans, several studies have reported a beneficial effect of an elevated n-3 PUFA intake and low omega-6/omega-3 ratio on bone health and osteoporosis (4,11–14). Omega-3 PUFA may protect against bone loss during menopause (15), decrease bone resorption (16,17), increase BMD at the vertebral level (13,18) and at the femoral neck (18) and decrease the risk of hip fracture (19). Conversely, in some studies, a high intake of omega-6 PUFAs was associated with a higher risk of fracture in the elderly (20).

To our knowledge, no one has investigated the relationship between PUFA intake and bone quality assessed by quantitative ultrasound (QUS) parameters. QUS parameters reflect the intrinsic quality and biomechanical properties of the bone; they provide complementary information to that of BMD on bone strength (21). Ultrasonography has several advantages for bone quality assessment (22) and may be the only technique applicable in remote and isolated communities, where BMD measurement by dual-energy x-ray absorptiometry (DXA) cannot be performed for osteoporosis diagnosis (23).

The aim of our study was to examine the relationship between the omega-3 and omega-6 PUFA contents of erythrocyte membrane phospholipids determined at baseline and QUS parameters [speed of sound (SOS) and broadband attenuation (BUA)] measured 2 years later at the calcaneus of Inuit women living in Nuuk (Greenland).

## Methods

### Population

A descriptive cross-sectional study was conducted from 12 to 20 September, 2000, to estimate bone strength and associated factors in peri-and postmenopausal Inuit women of Nuuk (Godthab, Greenland, 64°N parallel). Data collection was previously described in Côté et al. (24). The criteria for participating in the study were being female, aged between 49 and 64 years and born in Greenland. A random sample of 200 women was drawn from the list of Statistics Greenland, which included 547 potentially eligible women. Among the 200 women, 7 had died, 11 had moved and 15 were outside the city at the time of the study (for non-medical reasons). Finally, 167 women were invited to participate in the study and attend the Primary Health Care Clinic of Queen Ingrid's Hospital in Nuuk. Eight of them refused, 6 were excluded because of diseases (HIV, mental illness, influenza); hence 153 Inuit women participated in the study (24). Participants answered a questionnaire adapted from Mediterranean Osteoporosis Study administered by a Danish interviewer during a face-to-face interview (24). Subsequently, the women were invited to participate in a clinic session with blood collection, anthropometric measurements and QUS parameters measured at the right calcaneus. Out of the 153 women who had ultrasound measurements, 148 had enough plasma collected to allow for biochemical analyses.

Two years later, a longitudinal component was added to the original study, and from 26 August to 3 September, 2002, participants were invited to a second measurement performed using the same technique and the same instrument as in 2000. Of the 153 participants in 2000, 121 completed the study in 2002. Among the 32 non-participants in 2002, 3 had died, 5 had moved, 11 could not be reached by phone, 7 refused to participate and 6 did not show up at their appointment. Biochemical data were available for 118 out of the 121 Inuit women who had both QUS measurements.

The project was approved by the Commission for Scientific Research in Greenland, by the Ethical Committee of the Greenlandic Health Commission (Denmark) and by the Research Ethics Committee of Hospital Saint-François d'Assise (Quebec, Canada). Participation in the study was voluntary, and a consent form was signed by each participant. All information concerning the participants was kept strictly confidential.

### Measurements and analyses

#### Bone measurements

Ultrasound parameters (BUA and SOS) were measured at the right calcaneus participants with a water-bath portable ultrasound device Achilles Lunar (Lunar Corporation, Madison, WI, USA) (25). A research nurse calibrated the instrument daily using the acoustic phantom provided by the manufacturer. In vivo precision was assessed by repeated measurements conducted for 15 subjects: the mean coefficient of variation (CV) was 0.8% for BUA and 0.2% for SOS (24). In vivo measurements for a 42 year-old woman performed over a 1-year period led to CV values of 1.4% for BUA and 0.2% for SOS.

#### Anthropometric measurements

Weight (kg) and height (cm) were measured by research nurses using standardized techniques.

#### Laboratory analyses

Polyunsaturated fatty acids (omega-3 and omega-6 PUFAs) were measured in erythrocyte membrane phospholipids following transmethylation with a HP5890 gas chromatograph (Hewlett Packard, Toronto, ON) equipped with a HP8823 capillary column, a flame ionization detector (FID) and a HP7673A automatic injector. The analyses were performed at the Department of Nutritional Sciences, University of Guelph (Ontario, Canada) (24). Cholesterol and triglycerides concentrations (mmol/L) were determined by standard enzymatic procedures on an Auto-analyser II instrument (Technicon Instruments Corporation, Tarrytown, NY) (24).

## Questionnaire

The questionnaire collected socio-demographic information (date and place of birth, level of education: none or primary/secondary and higher), gynaecological history [menopausal status: postmenopausal/non-menopausal, hormone replacement therapy (HRT), yes/no; past use of oral contraceptives, yes/no], lifestyle habits (current smoker, yes/no; calcium supplements during the last month, yes/no; weekly fish consumption, <3 meals/≥3 meals) and personal history fractures during adulthood (yes/no). Women were considered postmenopausal if they had no menstrual period for 1 year before the date of entry in the study, or if they had a bilateral oophorectomy for more than 6 months and/or a level of follicle-stimulating hormone >40 IU/L.

## Statistical analyses

Analyses were performed on data from the 118 participants who had 2 QUS measurements (at baseline in 2000 and at follow-up in 2002). Descriptive statistics were presented for continuous variables (mean, standard deviation, minimum, maximum) and categorical variables (number and percent for each modality). Pearson correlation coefficients (r) were calculated between age, QUS parameters and PUFA contents.

Total contents of omega-3 PUFAs and omega-6 PUFAs were calculated and expressed as percentages of total fatty acids in erythrocyte membrane phospholipids. Omega-3 PUFAs include the following fatty acids: alpha-linolenic acid (ALA, C18:3n-3), stearidonic acid (STA, C18:4n-3), eicosatrienoic acid (C20:3n-3), eicosatetraenoic acid (C20:4n-3), eicosapentaenoic acid (EPA, C20:5n-3), docosapentaenoic acid (DPA, C22:5n-3), docosahexaenoic acid (DHA, C22:6n-3); the last 5 are highly polyunsaturated fatty acids (HPFA). Omega-6 PUFAs comprise the following fatty acids: linoleic acid (LA, C18:2n-6), gamma-linolenic acid (GLA, C18:3n-6), eicosadienoic (C20:2n-6), dihomo-gamma-linolenic acid (DGLA, C20:3n-6), arachidonic acid (AA, C20:4n-6), docosadienoic acid (C22:2n-6), adrenic acid (C22:4n-6) and osbond acid (OBA, C22:5n-6).

The relationships between PUFAs and both QUS parameters were examined using multiple linear regression models: 3 models were proposed that included different sets of covariates. Multicollinearity diagnostics were performed for all independent variables. For continuous variables, normality, linearity and homoscedasticity of residuals were tested graphically and by hypothesis testing.

The moderating effect of the main omega-6 PUFAs (LA, AA, OBA) on the relationship between omega-3 PUFAs (ALA, EPA, DHA) and QUS parameters was tested by introducing into the multiple regression models an interaction term between the specific omega-3 PUFA and omega-6 PUFA considered. Models were adjusted for several confounders and covariates. The QUS parameter value measured at baseline (year 2000) was part of the adjustment variables in models along with other factors measured at baseline.

A p-value<0.05 in the bilateral situation was considered statistically significant. The SAS version 9.2 software was used to conduct all statistical analyses (SAS Institute Inc., Cary, NC, USA).

## Results

### Participant characteristics

Characteristics of participants to both QUS measurements are listed in Table I. Most participants were postmenopausal, had a low level of education and were smokers. Few of them reported a history of fracture as an adult, or use of calcium supplements, HRT or oral contraceptives. The fractures reported were mostly wrist fractures; one woman with a wrist fracture also reported a vertebral fracture during adulthood resulting from a fall. No difference was observed between the characteristics of the 30 women who had only bone measurements at baseline and those of the 118 participants with baseline and follow-up QUS measures (26). Over the 2-year period, QUS parameters decreased in Inuit women: mean percent changes (±SD) were −0.31% (±0.69) for SOS values and −1.35% (± 5.95) for BUA values.

**Table I:** Characteristics of Greenlandic Inuit women

Characteristic	Mean±SD (N=118)	Range
2002 measurements		
SOS (m/s)	1518±23	1464–1600
BUA (dB/MHz)	106.4±10.6	80–144
2000 measurements		
SOS (m/s)	1523±23	1464–1595
BUA (dB/MHz)	108.0±9.6	84–136
Age (years)	55.3±4.2	49–64
Weight (kg)	67.6±14.5	37–99
Height (cm)	155.9±6.7	142–171
Total cholesterol (mmol/L)	2.6±0.5	1.7–4.7
Total triglycerides (mmol/L)	1.6±1.3	0.4–13.4
	N (%)	
Menopausal status	118	
Postmenopausal	107 (90.7)	
Non-menopausal	11 (9.3)	
Level of education	116	
None or primary school	109 (94.0)	
Secondary or higher	7 (6.0)	
Current smoker	118	
Yes	85 (72.0)	
No	33 (28.0)	
Calcium supplement use	118	
Yes	12 (10.2)	
No	106 (89.8)	
Personal history of fracture	118	
Yes	9 (7.6)	
No	109 (92.4)	
Fish consumption	118	
<3 meals/week	100 (84.8)	
≥3 meals/week	18 (15.2)	
Oral contraceptive use	118	
Yes	47 (39.8)	
No	71 (60.2)	
HRT use	118	
Yes	10 (8.5)	
No	108 (91.5)	

Contents of the various PUFAs in erythrocyte membrane phospholipids are presented in Table II. EPA, DHA and ALA represented respectively 35.4%, 52.4% and 1.9% of the total omega-3 PUFA content of erythrocyte membrane phospholipids. LA represented 65.3% AA, 23.7% and OBA, 0.5% of total omega-6 PUFA content.

**Table II:** Omega-3 and omega-6 PUFA contents of erythrocyte membrane phospholipids in Greenlandic Inuit women

PUFA^a^	N	Mean±SD	Range^b^
% of the total fatty acids			
C18:3n-3 (ALA)	117	0.27±0.11	0.02–0.71
C18:4n-3 (STA)	118	0.03±0.05	0.00–0.53
C20:3n-3	118	0.03±0.02	0.00–0.12
C20:4n-3	118	0.03±0.03	0.00–0.19
C20:5n-3 (EPA)	118	4.81±2.83	0.60–12.36
C22:5n-3 (DPA)	118	1.48±0.42	0.53–2.57
C22:6n-3 (DHA)	118	7.32±1.85	2.70–13.05
Σomega-3 HPFA^c^	118	13.67±4.69	4.27–27.32
Σomega-3 PUFA^d^	118	13.97±4.63	4.78–27.56
C18:2n-6 (LA)	118	14.89±4.08	5.94–25.41
C18:3n-6 (GLA)	118	0.11±0.04	0.01–0.22
C20:2n-6	118	0.50±0.17	0.08–0.91
C20:3n-6 (DGLA)	118	1.71±0.72	0.25–3.81
C20:4n-6 (AA)	118	5.41±1.23	3.50–9.29
C22:2n-6	118	0.02±0.02	0.00–0.13
C22:4n-6	118	0.06±0.06	0.00–0.28
C22:5n-6 (OBA)	118	0.11±0.07	0.00–0.32
Σomega-6 PUFA^e^	118	22.79±4.47	12.44–31.45
Ratio			
Σomega-3/Σomega-6 PUFA	118	0.68±0.37	0.17–2.21
ALA/LA	117	0.02±0.01	0.003–0.04
EPA/LA	118	0.41±0.40	0.03–1.92
DHA/LA	118	0.57±0.34	0.12–2.15
Σomega-3 HPFA/LA	118	1.11±0.79	0.19–4.50
EPA/AA	118	0.91±0.52	0.14–2.29
DHA/AA	118	1.42±0.47	0.43–2.62
Σomega-3 HPFA/AA	118	2.62±0.98	0.67–5.30

a Measured at baseline (year 2000).

b Minimum–maximum.

c Omega-3 HPFA=ΣC20:3n-3+C20:4n-3+C20:5n-3+C22:5n-3+C22:6n-3.

d Omega-3 PUFA=ΣC18:3n-3+C18:4n-3+C20:3n-3+C20:4n-3+C20:5n-3+C22:5n-3+C22:6n-3.

e Omega-6 PUFA=ΣC18:2n-6+C18:3n-6+C20:2n-6+C20:3n-6+C20:4n-6+C22:2n-6+C22:4n-6+C22:5n-6.

### Pearson's correlation coefficients

QUS values at follow-up were positively correlated to those at baseline (SOS_year 2002_ and SOS_year 2000_: r=0.89; BUA_year 2002_ and BUA_year 2000_: r=0.81; p<0.0001). SOS_year 2002_ and BUA_year 2002_ values were also intercorrelated (r=0.65, p<0.0001). SOS_year 2002_ but not BUA_year 2002_ was negatively correlated to age at baseline (Table S1). Total omega-3 PUFA content, as well as those of EPA, DHA, DPA and STA, were positively correlated with age, whereas total PUFA omega-6 content and those of LA and DGLA were negatively correlated with age at baseline (Table S1). EPA and DHA contents were positively correlated with the consumption of 3 or more fishmeals per week recorded at baseline (r=0.29, p=0.0018 and r=0.24, p=0.0077, respectively).

### Multivariate regression models

None of the main omega-6 PUFAs (LA, AA, OBA) had any modifying effect on the relationship between omega-3 PUFAs (ALA, EPA, DHA) and QUS parameters.

#### BUA_year 2002_

EPA, DHA, total HPFA or total omega-3 PUFA contents were positively and significantly associated with BUA_year 2002_, mainly in models adjusted for AA or OBA and other variables (Table III and Tables S2 and S3). EPA/LA, DHA/LA or HPFA/LA ratios were positively and significantly associated with BUA_year 2002_ in all multivariate models. Similarly, total omega-3/total omega-6 and EPA/AA ratios were positively and significantly associated with BUA_year 2002_ (Table III and Tables S2 and S3).

**Table III:** Multivariate models for SOS (m/s) and BUA (dB/MHz) measures performed in 2002

Main exposure variable		SOS^a^			BUA^b^		
			
PUFA	N	R^2^	Regression coefficient (SE)	p-value	R^2^	Regression coefficient (SE)	p-value
ALA^c^	115	0.8452	0.805 (9.999)	0.9360	0.7230	−4.506 (6.277)	0.4745
ALA^d^	115	0.8503	3.130 (8.622)	0.7173	0.7156	−9.942 (5.584)	0.0780
ALA^e^	115	0.8447	−2.199 (8.325)	0.7922	0.7156	−10.313 (5.299)	0.0544
ALA^f^	115	0.8473	5.850 (10.315)	0.5718	0.7207	−4.880 (6.545)	0.4576
EPA^c^	116	0.8451	0.276 (0.545)	0.6140	0.7311	0.654 (0.340)	0.0569
EPA^d^	116	0.8495	0.123 (0.343)	0.7205	0.7311	0.648 (0.218)	0.0036
EPA^e^	116	0.8451	0.277 (0.343)	0.4213	0.7325	0.685 (0.214)	0.0019
EPA^f^	116	0.8465	−0.106 (0.526)	0.8414	0.7312	0.695 (0.329)	0.0369
DHA^c^	116	0.8447	−0.053 (0.851)	0.9506	0.7226	0.364 (0.535)	0.4984
DHA^d^	116	0.8494	0.132 (0.525)	0.8023	0.7211	0.744 (0.338)	0.0297
DHA^e^	116	0.8444	0.243 (0.546)	0.6569	0.7212	0.824 (0.346)	0.0189
DHA^f^	116	0.8470	−0.473 (0.762)	0.5367	0.7220	0.472 (0.485)	0.3328
Σomega-3 HPFA^c^	116	0.8450	0.163 (0.404)	0.6870	0.7296	0.444 (0.251)	0.0799
Σomega-3 HPFA^d^	116	0.8495	0.073 (0.211)	0.7294	0.7296	0.384 (0.134)	0.0050
Σomega-3 HPFA^e^	116	0.8450	0.160 (0.214)	0.4563	0.7310	0.414 (0.134)	0.0025
Σomega-3 HPFA^f^	116	0.8467	−0.165 (0.367)	0.6533	0.7297	0.454 (0.230)	0.0506
Σomega-3 PUFA^c^	116	0.8449	0.153 (0.411)	0.7100	0.7292	0.440 (0.256)	0.0880
Σomega-3 PUFA^d^	116	0.8495	0.072 (0.214)	0.7361	0.7292	0.386 (0.136)	0.0054
Σomega-3 PUFA^e^	116	0.8450	0.159 (0.217)	0.4662	0.7307	0.417 (0.136)	0.0027
Σomega-3 PUFA^f^	116	0.8467	−0.176 (0.371)	0.6357	0.7293	0.450 (0.233)	0.0559
LA^g^	116	0.8467	−0.464 (0.435)	0.2884	0.7297	0.092 (0.273)	0.7375
LA^h^	116	0.8465	−0.362 (0.386)	0.3501	0.7312	0.039 (0.242)	0.8720
LA^i^	116	0.8470	−0.462 (0.356)	0.1973	0.7220	−0.192 (0.228)	0.4013
LA^j^	115	0.8473	−0.403 (0.309)	0.1943	0.7207	−0.270 (0.196)	0.1720
AA^g^	116	0.8495	1.410 (0.803)	0.0822	0.7296	0.114 (0.509)	0.8227
AA^h^	116	0.8495	1.399 (0.809)	0.0866	0.7311	0.061 (0.511)	0.9047
AA^i^	116	0.8494	1.450 (0.788)	0.0685	0.7211	0.314 (0.507)	0.5368
AA^j^	115	0.8503	1.613 (0.830)	0.0547	0.7156	0.097 (0.537)	0.8568
OBA^g^	116	0.8450	0.715 (14.159)	0.9598	0.7310	6.849 (8.870)	0.4418
OBA^h^	116	0.8451	0.613 (14.081)	0.9654	0.7325	6.380 (8.804)	0.4703
OBA^i^	116	0.8444	−0.037 (14.271)	0.9979	0.7212	5.984 (9.076)	0.5112
OBA^j^	115	0.8447	−1.666 (13.923)	0.9050	0.7156	1.754 (8.882)	0.8439
Σomega-6 PUFA^g^	116	0.8450	0.008 (0.453)	0.9864	0.7296	0.070 (0.282)	0.8036
Σomega-6 PUFA^h^	116	0.8451	0.001 (0.378)	0.9970	0.7311	−0.0001 (0.235)	0.9997
Σomega-6 PUFA^i^	116	0.8447	−0.167 (0.376)	0.6572	0.7226	−0.230 (0.237)	0.3355
Σomega-6 PUFA^j^	115	0.8452	−0.155 (0.283)	0.5856	0.7230	−0.294 (0.178)	0.1011
Omega-3/omega-6 PUFA^k^	116	0.8453	2.347 (2.633)	0.3748	0.7279	4.795 (1.652)	0.0045
EPA/LA^k^	116	0.8454	2.198 (2.400)	0.3619	0.7243	3.999 (1.517)	0.0097
DHA/LA^k^	116	0.8458	3.092 (2.927)	0.2934	0.7218	4.545 (1.859)	0.0162
Σomega-3 HPFA/LA^k^	116	0.8457	1.253 (1.229)	0.3105	0.7236	2.012 (0.778)	0.0111
EPA/AA^k^	116	0.8443	0.634 (1.842)	0.7315	0.7290	3.434 (1.152)	0.0036
DHA/AA^k^	116	0.8453	−1.798 (2.055)	0.3835	0.7088	1.397 (1.332)	0.2966
Σomega-3 HPFA/AA^k^	116	0.8442	−0.296 (1.010)	0.7704	0.7187	1.404 (0.642)	0.0310

a Model adjusted for: other PUFA (see notes ^c^ to ^k^), SOS_year 2000_, age, menopausal status, weight, height, smoking, level of education, calcium supplements use, HRT use, oral contraceptive use, personal history of fracture (all measurements at baseline, year 2000).

b Model adjusted for: other PUFA (see notes ^c^ to ^k^), BUA _year 2000_, age, menopausal status, weight, height, smoking, level of education, calcium supplement use, HRT use, oral contraceptive use, personal history of fracture (all measurements at baseline, year 2000).

c PUFA for adjustment: Σomega-6 PUFA.

d PUFA for adjustment: AA.

e PUFA for adjustment: OBA.

f PUFA for adjustment: LA.

g PUFA for adjustment: Σomega-3 HPFA.

h PUFA for adjustment: EPA.

i PUFA for adjustment: DHA.

j PUFA for adjustment: ALA.

k Model adjusted for the variables indicated in notes ^a^ or ^b^, and non-adjusted for another PUFA.

#### SOS_year 2002_

AA was positively and significantly associated with SOS_year 2002_, but only in models adjusted for omega-3 PUFAs (EPA, DHA, total omega-3 HPFA or total omega-3 PUFA) and age, height, weight, menopausal status, smoking status, supplementation, education level, total cholesterol and total triglycerides (Model II, Table S3). No other PUFA was associated with SOS_year 2002_.

### Factors significantly associated with QUS parameters

In BUA_year 2002_ multivariate models (Table III), among confounders and covariates used for adjustment, 3 variables measured at baseline (year 2000) were constantly found to be associated with the dependant variable: BUA and HRT use (positively associated) and smoking (negatively associated). In SOS_year 2002_ multivariate models (Table III), 4 variables measured at baseline (year 2000) were constantly found to be associated with the dependant variable: SOS and HRT use (positively associated), smoking and height (negatively associated). In some models, age at baseline was also negatively and significantly associated with SOS_year 2002_ (data not shown).

## Discussion

To our knowledge, this is the first longitudinal study investigating the relationship between PUFA (omega-3 and omega-6) status and bone quality assessed by QUS parameters. In this group of aboriginal women consuming large amounts of marine species, the omega-3 PUFA content of erythrocyte membrane phospholipids measured at baseline was positively and significantly associated with BUA measured after a 2-year follow-up, in models adjusted for omega-6 PUFAs and several other variables. BUA is strongly related to structural parameters (27), the microarchitecture and orientation of trabeculae (28,29), reflecting the intrinsic quality of the bone. Lowest BUA values may suggest lower BMD values at different skeletal sites (29) and in turn, an increased risk of fragility fracture.

Few data currently exist on bone strength measured by ultrasound among Inuit. In the descriptive cross-sectional study of Côté et al., mean values for SOS and BUA measured at the right calcaneus of Inuit peri- and postmenopausal from Nuuk were, respectively, 1.4 and 4.0% lower than those measured with the same instrument in Caucasian women from Southern Quebec (n=2972, mean age=55.8 years) (24).

However, several studies have investigated the relationship between PUFA dietary intake or supplementation and BMD at different skeletal sites, or the risk of osteoporosis and fragility fractures. Generally, the results suggest a beneficial role of omega-3 PUFAs and a higher omega-3/omega-6 ratio on bone health. For example, it was reported that the omega-3 PUFA content of erythrocyte membrane phospholipids and fish consumption were positively correlated to bone mass in postmenopausal Korean women; a higher EPA+DHA level was associated with a lower risk of osteoporosis in these participants (14). Fish consumption (≥3 times/week) was associated with maintenance of BMD at the femoral neck in aged women and men from the *Framingham Osteoporosis Study* (30). Omega-3 PUFA supplementation significantly decreased bone resorption (as measured by urinary pyridinoline level) without affecting bone formation, 6 months after the initiation of treatment in postmenopausal osteoporotic Iranian women participating in a randomized clinical trial (17). Results from another randomized study suggested that supplementation with GLA and EPA in older postmenopausal women (mean age=79.5 years) with low bone mass, had beneficial effects on BMD at vertebral and femoral levels compared to the placebo group (18). An increase in the omega-6/omega-3 PUFA ratio was associated with low BMD at the hip in men and women aged 40–90 years who were participants in the *Rancho Bernardo Study* (31).

The average omega-3 PUFA content of erythrocyte membrane phospholipids in Inuit women from Nuuk (13.97%) was higher than that of Inuit women from Nunavik (11.93%) and Cree women from East James Bay (6.85%); mean omega-3/omega-6 PUFA ratios in these groups were 0.68, 0.54 and 0.23, respectively (26). By comparison, non-Aboriginal women with a Western-style diet show a higher omega-6 PUFA intake and a lower omega-3/omega-6 ratio (32).

Given the low rate of conversion of ALA to EPA and especially DHA in humans (33), the diet must provide the amounts required for normal cell function, with fatty fish and fish oil being major sources. Omega-3 PUFAs induce anti-inflammatory effect on bones, leading to a decrease in pro-inflammatory cytokines (interleukin-1, interleukin-6 and tumour necrosis factor-alpha) that play a critical role in bone renewal (34) and are involved in osteoporosis (11).

EPA and DHA are also substrates for the production of resolvins (E-and D-series) and protectins that are biologically active compounds (35). *In vitro* and laboratory animal studies have reported anti-inflammatory, inflammation-resolving and immunomodulatory effects of resolvins (36). EPA is also a precursor of anti-inflammatory eicosanoids (37). The production of prostaglandin E2 (PGE2), the main prostaglandin with bone resorption activity, and leukotrienes E4 (LTE4) by human inflammatory cells can be decreased significantly through supplementation with fish oil over weeks or months (38).

Another mechanism by which omega-3 PUFAs may influence bone quality is the regulation of intestinal calcium absorption through modulation calcium ATPase activity. DHA and EPA may decrease urinary excretion and increase calcium absorption in the duodenum, especially when dietary calcium is low (34). These properties may be especially important in Inuit who generally have a low dietary calcium intake.

The present study has several strong points. Firstly, its longitudinal design, with exposure factors determined 2 years before QUS parameters, strengthens a cause and effect relationship. Secondly, the type of sampling and recruitment allows us to generalize the results to all Inuit women aged 49–64 years who are residents of Nuuk (Greenland). Thirdly, we took into account a large number of adjustment factors known to be associated with QUS parameters in our multivariate models. However, residual confounding cannot be excluded. Determinants of bone strength are multifactorial and multigenic and other factors (genetic, nutritional, environmental) not measured in our study could explain some of the residual variance.

The main limitation of our study is that it was not designed from the outset as a longitudinal study and therefore the sample size was not planned to account for women lost to follow-up over the 2-year period. The percentage of participants lost to follow-up was important (20%), which can potentially lead to a selection bias. However, in our study, being lost to follow-up was neither related to PUFA status nor to bone strength. Women who did not have bone measurements in 2002 were not different from those who participated in both measures for all factors investigated.

In conclusion, we observed that BUA—a QUS parameter reflecting bone intrinsic quality and strength—increased with the content of long-chain omega-3 PUFAs and with a higher total omega-3/total omega-6 ratio in erythrocyte membrane phospholipids of Greenlandic Inuit women living in Nuuk. The frequent consumption of fatty fish and marine mammal fat provides intakes of EPA and DHA that favour bone health in these Inuit women.
